# Perinatal Exposure to Tartrazine Triggers Oxidative Stress and Neurobehavioral Alterations in Mice Offspring

**DOI:** 10.3390/antiox9010053

**Published:** 2020-01-08

**Authors:** Gadah Albasher, Najla Maashi, Saleh Alfarraj, Rafa Almeer, Tarfa Albrahim, Fatimah Alotibi, May Bin-Jumah, Ayman M. Mahmoud

**Affiliations:** 1 Department of Zoology, College of Science, King Saud University, Riyadh 11451, Saudi Arabia; galbeshr@ksu.edu.sa (G.A.); mashinajla@gmail.com (N.M.); alfarraj@ksu.edu.sa (S.A.); ralmeer@ksu.edu.sa (R.A.); 2 Clinical Nutrition, Department of Health sciences, College of Health and Rehabilitation Sciences, Princess Nourah Bint Abdulrahman University Riyadh, Riyadh 84428, Saudi Arabia; tiAlbrahim@pnu.edu.sa; 3 Department of Botany and Microbiology, College of Science, King Saud University, Riyadh 11451, Saudi Arabia; flotibi@ksu.edu.sa; 4 Department of Biology, College of Science, Princess Nourah bint Abdulrahman University, Riyadh 84428, Saudi Arabia; mnbinjumah@pnu.edu.sa; 5 Physiology Division, Zoology Department, Faculty of Science, Beni-Suef University, Beni-Suef 62514, Egypt

**Keywords:** azo dyes, neurotoxicity, food colorants, oxidative stress, E102

## Abstract

The use of synthetic azo dyes as coloring agents in food products has dramatically increased. This study evaluated the effect of perinatal exposure to tartrazine (TZ) on mice offspring, focusing on neurobehavioral alterations and oxidative stress. The female mice received TZ (2.5 and 5 mg/kg) via oral gavage during pregnancy and the first 15 days after birth. At days 21 and 35 after birth, male mice were sacrificed, and samples were collected for analyses. Perinatal exposure to TZ triggered tissue injury evidenced by the histological alterations and neuronal damage in the cerebrum, medulla oblongata, and cerebellum. TZ provoked lipid peroxidation and diminished cellular antioxidants in different brain regions of the newborns. In addition, TZ increased hemoglobin content, as well as erythrocytes, leukocytes, and platelets count at days 21 and 35 after birth. Both the locomotor behavior and anxiety reflex were significantly altered in mice exposed to TZ. In conclusion, perinatal exposure to TZ within an adequate daily intake range induced oxidative stress and neurobehavioral and hematological alterations in mice offspring. Therefore, consuming foods containing TZ during pregnancy and lactation warrants public awareness.

## 1. Introduction

Colorants are widely used to improve the sensory attributes and esthetic quality of products in the food and beverage industries. Colorants include the naturally occurring pigments and synthetic azo dyes [1]. Given their attractive coloring properties, variable hues, stability, and low cost, approximately eight million tons of the synthetic food colorants are produced every year [2]. The azo dyes have been receiving significant consideration since these synthetic agents can pose a health risk and exert negative effects on the liver, kidney, and nervous system [3,4,5,6]. Recent reports have pointed to the ability of azo dyes to elicit oxidative stress [4,5] and pro-inflammatory effects [1]. The toxic effect of azo dyes has been attributed to the aromatic amines produced from the cleavage of the aryl-N=N-aryl group by the intestinal microbiota [7]. These aromatic amines are thought to exert toxic, mutagenic, and carcinogenic effects [8].

Tartrazine (TZ, E102), a synthetic azo dye with lemon yellow color, is one of the most commonly used food colorants. TZ is used in food products, such as soft and sport drinks, jellies, sauces, and chewing gums, and several non-food consumables, including cosmetics, and soaps [9]. In addition, TZ is used as a cheap alternative for saffron in some countries [10]. The Joint FAO/WHO Expert Committee has established an acceptable daily intake (ADI) of 0 to 7.5 mg/kg body weight TZ per day [11]. Several studies have demonstrated the impact of TZ in experimental animals. For instance, administration of 700 mg/kg of TZ triggered cell growth dysregulation and hemorrhage but not lipid peroxidation (LPO) in the brain of adult rats [3]. Meyer et al. tested the hypothesis that TZ can cause periportal injury to the liver of mice exposed to 50 mg/kg/day for two weeks [5]. TZ induced periportal recruitment of inflammatory cells and mild periportal fibrosis in the exposed mice [5]. At doses of 10 and 100 mg/kg, TZ provoked oxidative damage in the liver and kidney of rats [6]. Additionally, the adult rodents that received 125 to 500 mg/kg TZ for 30 days showed alterations in learning and memory functions, and declined antioxidant defenses [12]. Although the doses of TZ used in these studies were higher than the recommended ADI which could explain the observed toxic effects, rats that received 7.5 mg/kg/day of TZ from weaning to the age of 12 months showed inflammatory cells infiltration in the gastric mucosa [7]. Furthermore, the chronic administration of 7.5 mg of TZ per kg diet for 90 days resulted in oxidative stress and liver injury in adult rats [4]. Therefore, oxidative stress is implicated in the mechanism of TZ toxicity in adult animals.

Children are the main consumers of colored foods and beverages, and therefore are more vulnerable to the effects of synthetic colorants. Accordingly, a randomized double-blind trial showed hyperactivity in three- and eight-to-nine-year-old children consuming foods containing artificial coloring agents [13]. In the same context, the intake of synthetic food colorants during pregnancy can provoke neurobehavioral changes and teratogenicity in the newborns. Recently, a reduction in the fetal weight, cardiomegaly, and liver and kidney injury were observed in rat newborns exposed to 4.5 mg/kg of TZ at the sixth to 15th day of gestation [14]. In addition, rat dams exposed to 1% and 2% dietary TZ during gestation and lactation and three months after weaning showed normal development and no adverse behavioral or physical effects except a small transient change in neuromotor clinging ability of the female newborns and a slight increase in red blood cells (RBCs) and hemoglobin (Hb) [15]. These differences could be explained by the different doses of TZ, route of supplementation, duration, and other factors. Therefore, this study evaluates the impact of perinatal exposure to TZ, administered at doses within the ADI range, on the mice offspring, with an emphasis on neurobehavioral alterations and redox imbalance.

## 2. Materials and Methods

### 2.1. Experimental Design

Fifteen male and 45 female 10-week old Swiss white mice weighing 25 to 30 g, obtained from the college of Pharmacy, King Saud University, were housed under standard conditions and supplied chow diet and water ad libitum. All experiments were approved by the Ethics Committee of the King Saud University (KSU-843620319).

The mice were exposed to male pheromones to increase the mating ratio. After exposure, vaginal swabs were obtained from the female mice to confirm the estrus stage. Three proestrus females and one male were housed in a mating cage for 12 h. The pregnant females were allocated into 3 groups, each contained 4 mice. One group was kept as a control, while the other 2 groups received 2.5 and 5 mg/kg TZ from the day one of pregnancy to day 15 after birth. TZ was dissolved in distilled water and administered via oral gavage. The doses of TZ were selected to fall within the ADI range. The control group received distilled water.

At days 21 and 35, six male mice from each experimental group were sacrificed, and blood and brain samples were collected. Samples from different brain regions were collected on 10% neutral buffered formalin, whereas others were stored at −80 °C.

### 2.2. Behavioral Study

#### 2.2.1. Locomotor Behavior

The locomotor activity of the control and TZ-induced mice was evaluated at day 25 after birth, as previously described by [16]. In a 30 × 80 × 80 cm arena, the number of washes, wall rears and squares crossed, and duration of mobility and immobility were determined over 300sec.

#### 2.2.2. Fear and Anxiety Reflex

Using the elevated plus maze (EPM) test, we evaluated the fear and anxiety reflex in the mice offspring at day 35 after birth [17]. The number of entries and time spent to explore the EPM arms were determined over a duration of 300 sec.

### 2.3. Determination of Malondialdehyde (MDA) and Antioxidants

Cerebrum, cerebellum, and medulla oblongata samples were homogenized in cold phosphate buffered saline (10% *w/v*), centrifuged at 1000 rpm, and the supernatant was collected. MDA was determined in the homogenates following the method of Ohkawa et al. [18]. Reduced glutathione (GSH, [19]) and superoxide dismutase (SOD, [20]) were also assayed in the homogenates.

### 2.4. Histological Investigation

The samples fixed 10% neutral buffered formalin for 24 h were processed for paraffin embedding. After cutting, the 5 μm sections were stained with hematoxylin and eosin (H&E) and examined using a Nikon microscope (Eclipse E200-LED, Tokyo, Japan).

### 2.5. Determination of Hematological Parameters

Hemoglobin (Hb) content, and the numbers of erythrocytes (RBCs), leukocytes (WBCs), and platelets were assayed in the blood of the newborn mice at days 21 and 35 using an automated hematology analyzer (Genius Kt-6400, Diamond Diagnostics Inc., Holliston, MA, USA).

### 2.6. Statistical Analysis

The results were presented as means ± standard error of the mean (SEM). All statistical comparisons were performed using one-way ANOVA followed by Tukey’s post hoc test. Differences were considered significant at *p* < 0.05.

## 3. Results

### 3.1. Effect of TZ on the Histology of Cerebrum, Cerebellum, and Medulla Oblongata

**Figure 1:**
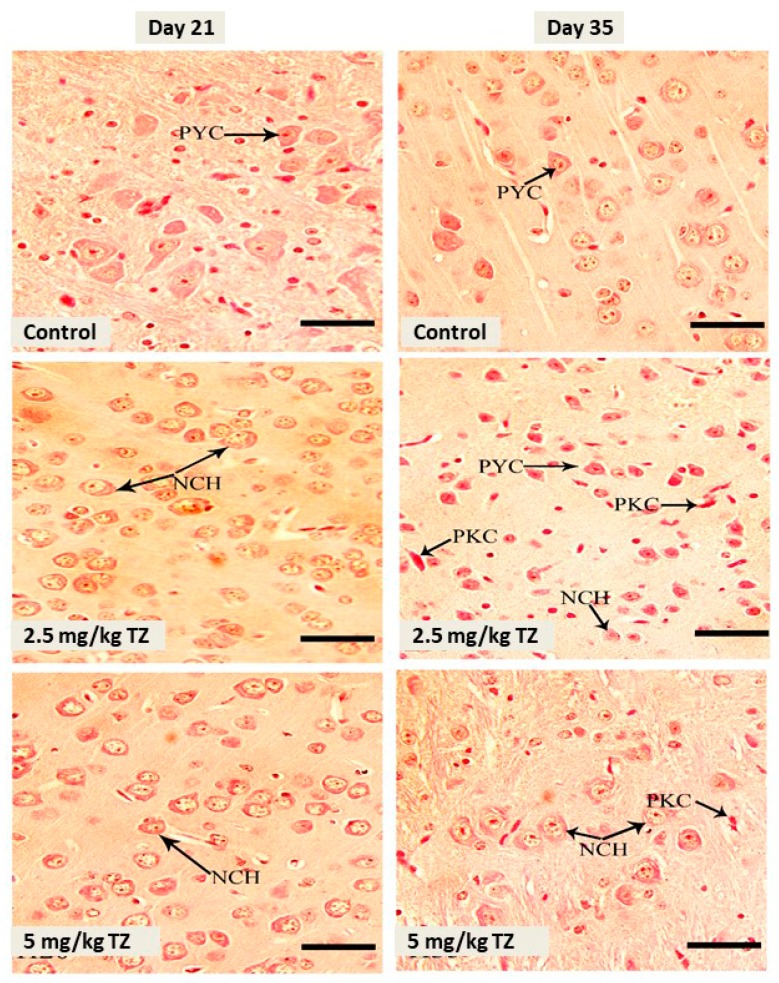
H&E stained sections in the cerebral cortex of mice newborns at days 21 and 35. The control mice showed normal histological structure and normal pyramidal cells (PYC), whereas perinatal exposure to tartrazine (TZ) induced neuronal chromatolysis (NCH) and pyknosis (PKC). Scale bar = 50 μm.

Mice newborns were sacrificed at days 21 and 35, and the brain samples were collected for examination. The cerebral cortex of the control newborns revealed normal structure, maturation, and distribution of neurons (Figure 1). Mice offspring exposed to 2.5 mg/kg and 5 mg/kg of TZ showed cerebral neuronal degeneration, pyknosis, and chromatolysis at days 21 and 35 (Figure 1).

**Figure 2:**
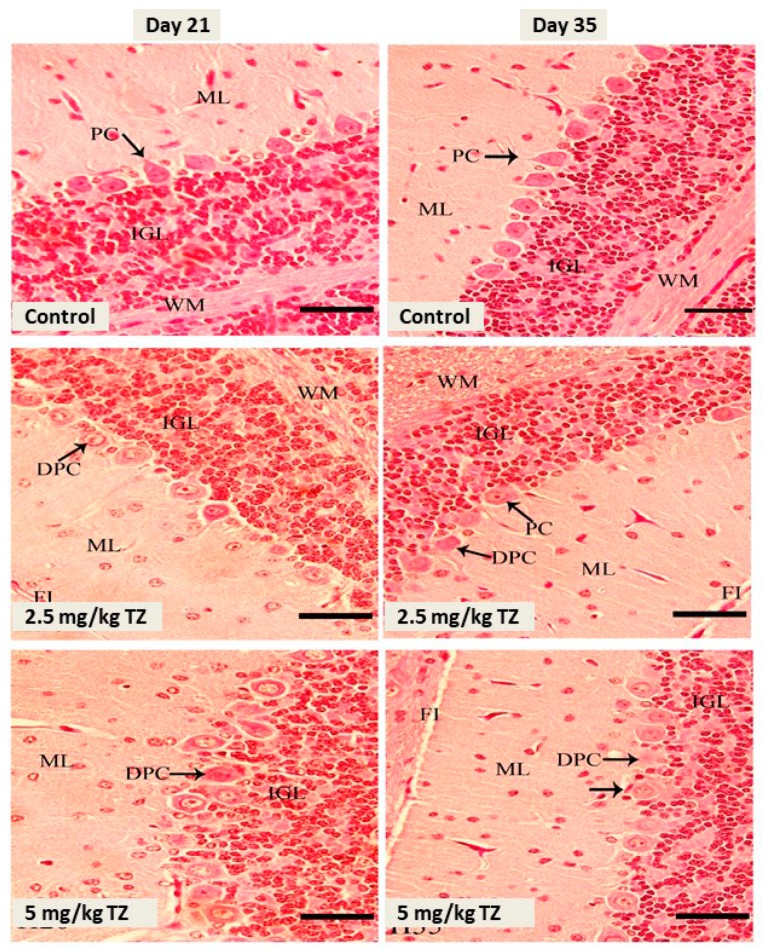
H&E stained sections in the cerebellar cortex of mice newborns at days 21 and 35. The control mice showed normal Purkinje cells (PC), fissure (FI), internal granular layer (IGL), molecular layer (ML), and white matter (WM). Perinatal exposure to TZ triggered histological alterations, including degenerated Purkinje cells (DPC). Scale bar = 50 μm.

Examination of the cerebellar cortex sections showed no histological alterations and normal granular layer, molecular layer, and Purkinje cells in the control newborns, as depicted in Figure 2. Conversely, mice exposed to either dose of TZ showed Purkinje cells degeneration, in particular with the high dose of TZ (Figure 2).

**Figure 3:**
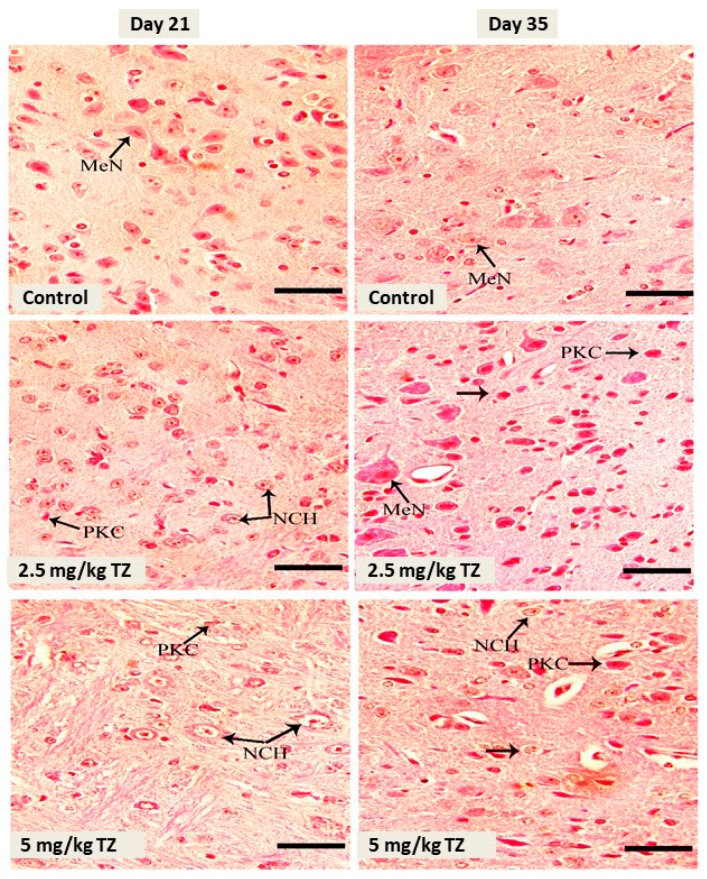
H&E stained sections in the medulla oblongata of mice newborns at days 21 and 35. The control mice showed normal medullary neurons (MeN). Perinatal exposure to TZ triggered histological alterations, such as neuronal chromatolysis (NCH) and pyknosis (PKC). Scale bar = 50 μm.

The control newborns showed normal medullary neurons, as revealed in Figure 3. Perinatal administration of either dose of TZ triggered neuronal pyknosis and chromatolysis at days 21 and 35.

### 3.2. TZ Induces Anxiety-Like Behavior in Newborns

**Figure 4:**
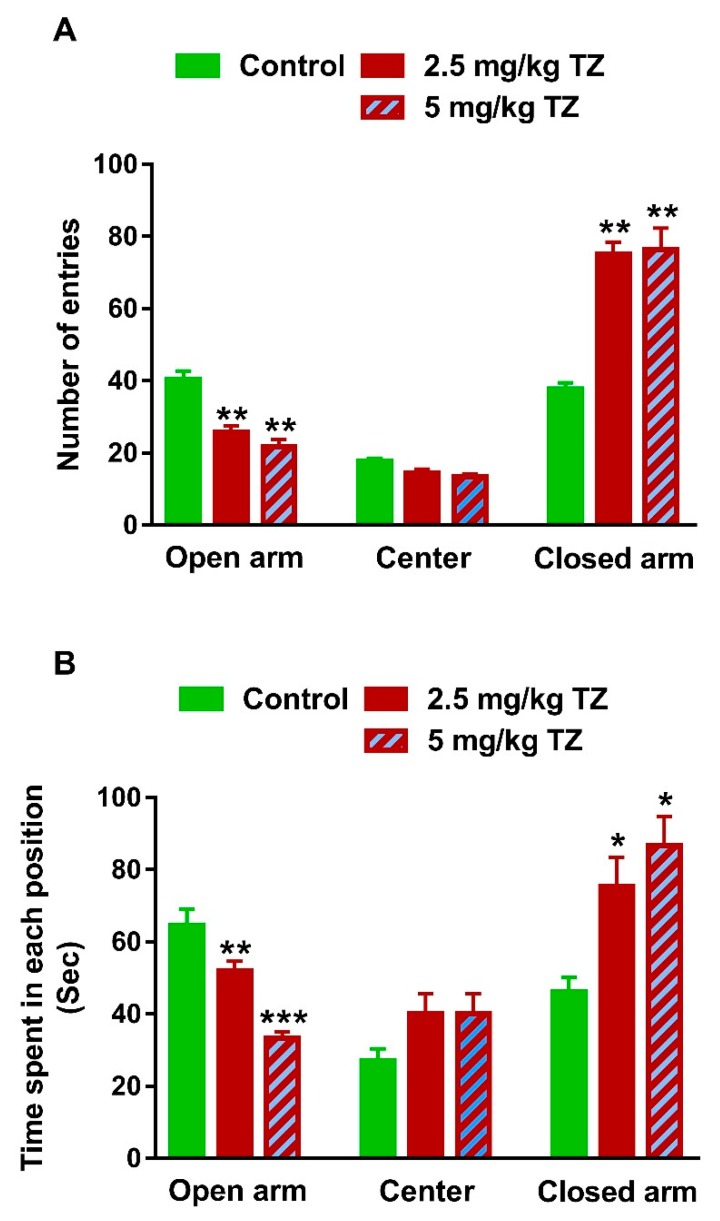
TZ-induced anxiety in mice newborns. (**A**) The newborns exposed to TZ showed a decrease in the number of entries to the open arm and increased number of entries to the closed arm of the EPM. (**B**) TZ-induced mice spent shorter time in the open arm and longer time in the closed arm of the EPM than the control newborns Data are mean ± SEM, (*n* = 6). * *p* < 0.05, ** *p* < 0.01, and *** *p* < 0.001 versus control group.

The EPM was used to assess the anxiety-like behavior [21]. The control and treated newborns were examined for fear and anxiety at day 35. The newborns exposed to 2.5 mg/kg and 5 mg/kg of TZ showed a decrease in the number of entries to the open arm, whereas the number of entries to the closed arm was increased, as represented in Figure 4A (*p* < 0.01).

The time spent in each arm of the elevated plus maze was recorded over 300 sec for every mouse. The time spent for the mice exposed to TZ in the open arm was shorter, whereas the time in the closed arm was significantly longer than that of the control newborns (Figure 4B). Of note, nonsignificant changes in both number of entries (Figure 4A) and time spent in the center (Figure 4B) were observed.

### 3.3. Effect of TZ on the Locomotion Behavior in Newborn Mice

**Figure 5:**
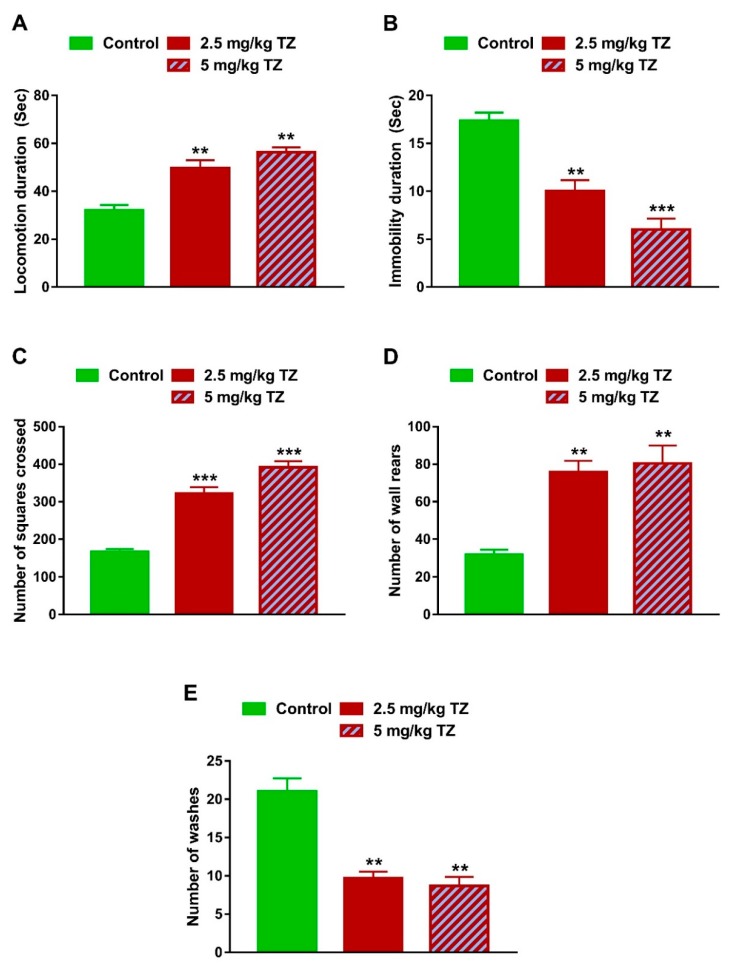
Effect of TZ on the locomotor behavior in newborn mice. Locomotion duration (**A**), number of squares crossed (**C**) and number of wall rears (**D**) were increased, and immobility duration (**B**) and number of washes (**E**) were decreased in newborns exposed to TZ. Data are mean ± SEM, (*n* = 6). ** *p* < 0.01 and *** *p* < 0.001 versus control group.

Newborn mice exposed perinatally to 2.5 mg/kg and 5 mg/kg of TZ had increased locomotion duration (*p* < 0.01 and *p* < 0.01, Figure 5A) and decreased immobility duration (*p* < 0.01 and *p* < 0.001, Figure 5B). Similarly, mice exposed perinatally to 2.5 and 5 mg/kg of TZ exhibited an increase in the number of squares crossed (*p* < 0.001 and *p* < 0.001, Figure 5C) and number of wall rears (*p* < 0.01 and *p* < 0.01, Figure 5D), whereas the number of washes was reduced (*p* < 0.01 and *p* < 0.01, Figure 5E).

### 3.4. TZ Induces LPO and Suppresses Antioxidants in the Brain of Newborn Mice

**Figure 6:**
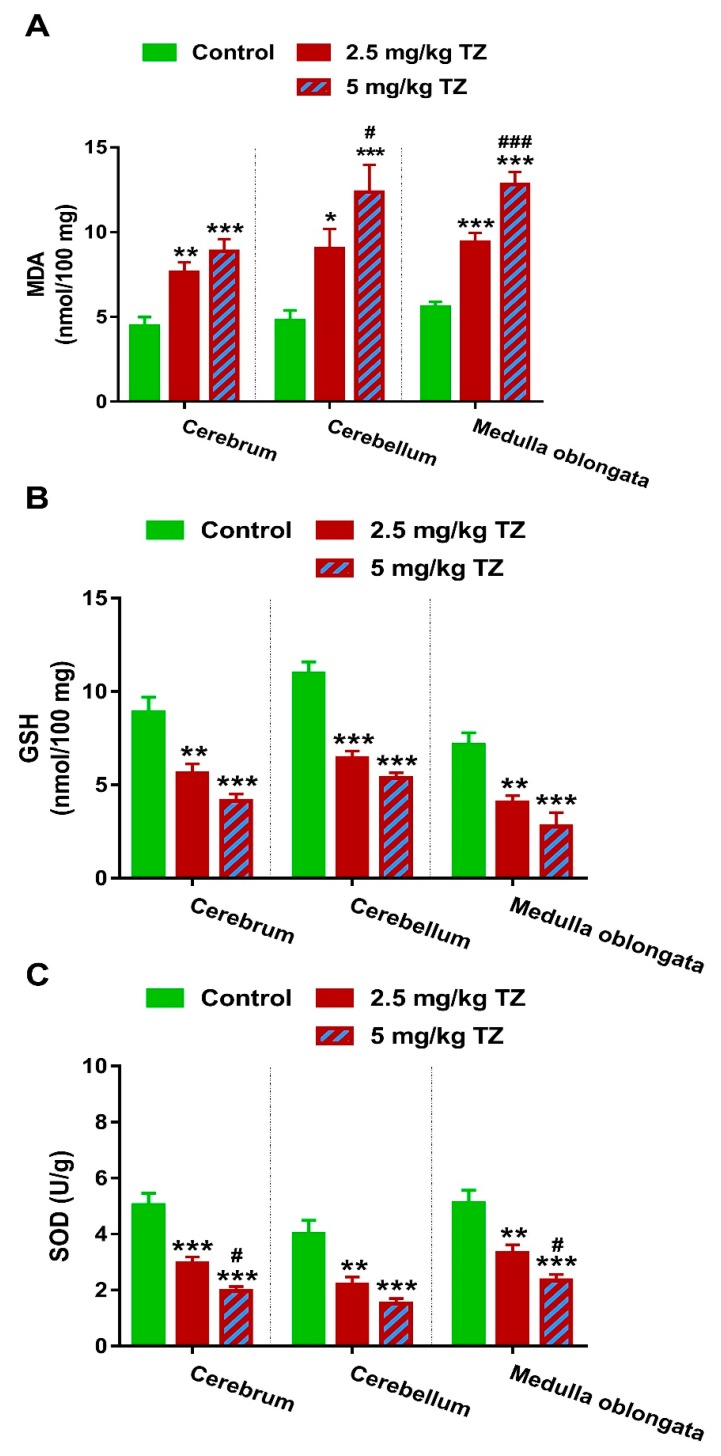
TZ induces oxidative stress in the cerebrum, cerebellum, and medulla oblongata of the newborn mice. Perinatal exposure to TZ provoked lipid peroxidation (**A**) and decreased GSH (**B**), and SOD (**C**) in different brain regions. Data are mean ± SEM, (*n* = 6). * *p* < 0.05, ** *p* < 0.01, and *** *p* < 0.001 versus control group and # *p* < 0.05 and ### *p* < 0.001 versus 2.5 mg/kg TZ.

Perinatal exposure to 2.5 and 5 mg/kg of TZ triggered LPO in the cerebrum (*p* < 0.01 and *p* < 0.001), cerebellum (*p* < 0.05 and *p* < 0.001) and medulla oblongata (*p* < 0.001 and *p* < 0.001) of the newborn mice (Figure 6A). In contrast, GSH was reduced significantly in the cerebrum (*p* < 0.01 and *p* < 0.001), cerebellum (*p* < 0.001 and *p* < 0.001) and medulla oblongata (*p* < 0.01 and *p* < 0.001) of newborns exposed to 2.5 mg/kg and 5 mg/kg of TZ (Figure 6B). Both TZ doses reduced SOD activity significantly in all studied brain regions (Figure 6C). Of note, TZ exerted a dose-dependent effect on MDA levels in the cerebellum and medulla oblongata, and SOD activity in the cerebrum and medulla oblongata.

### 3.5. TZ Alters Hematological Parameters in Newborn Mice

**Figure 7:**
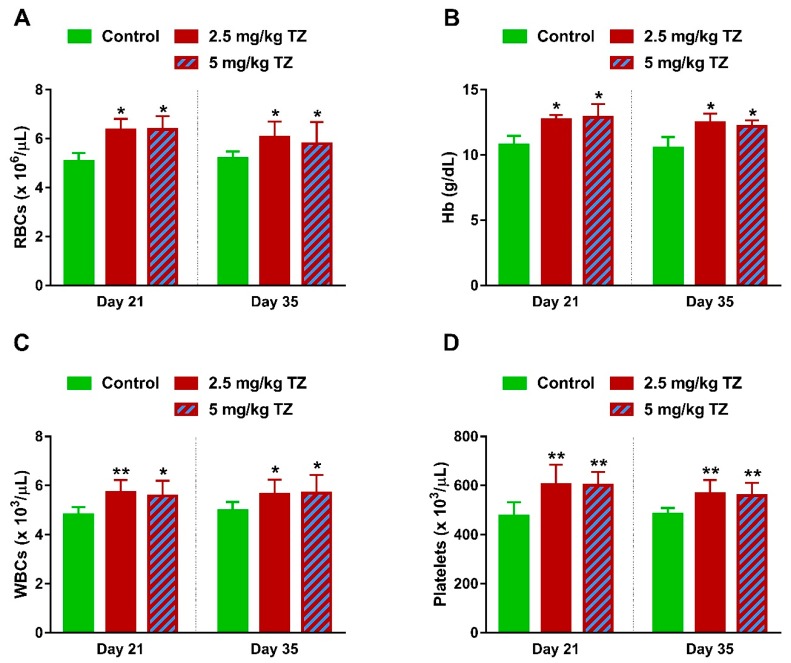
Effect of TZ on hematological parameters in newborn mice. Perinatal exposure to TZ increased (**A**) red blood cells (RBCs), (**B**) hemoglobin (Hb), (**C**) leukocytes (WBCs), and (**D**) platelets in the newborn mice at days 21 and 35 after birth. Data are mean ± SEM, (*n* = 6). * *p* < 0.05 and ** *p* < 0.01 versus control group.

Mice offspring exposed to TZ showed a significant increase in RBCs count (Figure 7A) and Hb (Figure 7B) at days 21 and 35 after birth. WBCs and platelets were increased significantly at days 21 and 35, in mice exposed to TZ (Figure 7C,D, respectively).

## 4. Discussion

The food additive dye, TZ, induced oxidative stress in the liver and brain [3,4,5], altered learning and memory functions [12], and triggered neurobiochemical alterations [22] in adult rodents. In this study, we investigated the effect of perinatal exposure to TZ on the mice newborns, focusing on the behavioral alterations and oxidative stress.

Exposure to TZ within the ADI range triggered tissue damage in different brain regions of the newborns. Although the hazardous effects of TZ have been demonstrated in adult rodents, few studies have scrutinized its impact on the neonates. Recently, newborns of rats exposed to TZ (4.5 mg/kg) at the sixth to 15th day of gestation showed a decrease in fetal weight and length, cardiomegaly along with liver and kidney injury [14]. These effects could be explained in terms of mutagenicity provoked by exposure to food additives [23]. In this study, perinatal exposure to 2.5 and 5 mg/kg of TZ resulted in pyknosis, chromatolysis, and degeneration of cerebral, cerebellar, and medullary neurons. The deleterious effects of TZ on the brain of adult rats and mice have been demonstrated in multiple studies. Oral administration of 7.5 mg/kg TZ for 40 consecutive days in rats resulted in oxidative stress in the cerebellum, striatum, frontal cortex, and hippocampus, evidenced by increased LPO and declined antioxidants [22]. Alsalman et al. recently reported cerebral apoptosis and histological alterations in rats challenged with 700 mg/kg of TZ for two weeks [3]. In addition, adult rats and mice that received 125 to 500 mg/kg of TZ for one month exhibited cerebral oxidative damage and apoptosis [12]. However, the doses of TZ used in both studies were approximately 16- to 93-fold higher than the recommended ADI and the authors did not specify which brain region they targeted. Furthermore, oral administration of 15 and 100 mg/kg of TZ caused hepatic and renal oxidative stress in rats [6]. Therefore, oxidative stress represents one of the main mechanisms underlying the toxicity of TZ.

Given the reported oxidative stress in different tissues of TZ-induced adult rats and mice, we assumed that redox imbalance is implicated in brain damage induced by perinatal exposure to this azo dye. The brain is liable to oxidative damage due to its high content of polyunsaturated fatty acids. Accordingly, exposure to TZ elicited LPO in different brain regions of the newborns. The redox imbalance was confirmed by the diminished cerebral, cerebellar, and medullary GSH content and SOD activity. Although the exact mechanisms of their toxicity are not fully elucidated, previous studies have pointed to the role of aromatic amines produced during the metabolism of azo compounds. The presence of the N=N functional group and aromatic rings leads to the production of aromatic amines which can possess mutagenic and carcinogenic effects [8]. TZ is metabolized by the gastrointestinal microflora to produce sulphanilic acid [7] which can diffuse across the blood-brain barrier (BBB) and enter the brain of developing rats [24]. Aromatic amines have been identified in the urine of experimental animals that received azo dyes and the dyestuff workers [25]. Various azo dye products have been reported to exert toxic effects only following reduction by intestinal microbiota [26], with increased reactive oxygen species (ROS) levels which represent the main culprit behind their toxicity. Our results added support to the previous studies by suggesting the role of ROS in mediating the toxicity of azo dyes and highlighted the implication of oxidative stress in neurotoxicity induced by perinatal exposure to TZ.

The impact of TZ on the nervous system was further investigated via assessment of the fear and anxiety reflex and locomotor activity. Recently, we reported the correlation between oxidative stress triggered by perinatal exposure to different agents and neurobehavioral alterations in mice newborns [27,28]. Here, the newborns exposed to TZ needed a shorter time to explore the open arm of the maze, whereas the time taken in the closed arm was longer than that of the control mice, demonstrating anxiety and fear. The developed anxiety in the newborn mice could be explained in terms of TZ-induced oxidative stress. The relationship between high anxiety levels and disorders and oxidative stress has been reviewed by Bouayed et al. [29]. Moreover, the locomotion behavior was assessed and the result showed an increased locomotion time in mice exposed to TZ. The assessment of the locomotor behavior was used in the initial screening of the pharmacological effects of drugs and did not involve extensive conditioning or learning [30]. Therefore, the altered locomotor behavior of the newborn mice pointed to the deleterious effects of TZ. In support of these findings, consumption of foods containing artificial coloring agents has affected childhood behavior and resulted in hyperactivity in children [13]. 

Next, we evaluated the effect of TZ on RBCs, WBCs and platelets count, and Hb content in the newborn mice. Hematological parameters have been demonstrated to be altered following exposure to toxic agents [31,32,33,34] as well as in metabolic diseases [35]. Therefore, estimation of the hematological parameters can represent a powerful tool and earlier indicator to assess the detrimental effects of drugs, chemicals, and other agents. In this study, perinatal exposure to TZ triggered an increase in the circulating numbers of RBCs, WBCs and platelets, and Hb content. Accordingly, the administration of azo dyes for 90 days increased RBCs and WBCs in adults rats, while platelets were not affected [36]. Our findings were further supported by the study of Sobotka et al. in which a slight increase in RBCs and Hb was observed in rat dams exposed to 1% and 2% dietary TZ during gestation and lactation and for three months after weaning [15]. The increase in RBCs count could be a response to low oxygen supply to the tissues due to the toxic effect of TZ. Leo et al. recently demonstrated the ability of azo dyes, including TZ, to provoke pro-inflammatory responses in vitro [1], and this could explain the increased WBCs count in our study. The increased WBCs count could be the result of an activated immune system in response to tissue damage [36]. In addition to leukocytosis, mice exposed to TZ showed thrombocytosis. Accordingly, the number of platelets increased in adults that received the azo dye carnosine for 120 days, as recently reported by [37]. The authors attributed the increase in platelets to the immune response and supported their hypothesis by the notion that platelets act as circulating sensors linking tissue repair and immune responses [38].

## 5. Conclusions

These results confer information that perinatal exposure to the azo dye TZ, within the ADI range, induced neurobehavioral alterations in mice newborns. TZ triggered histological alterations and oxidative stress in different regions of the brain. In addition, perinatal exposure to TZ caused hematological alterations, altered the locomotor behavior, and induced anxiety-like behavior in the newborn mice. Although it was administered within the ADI range, the use of TZ during pregnancy and lactation can pose teratogenic effects and neurobehavioral alterations in newborn mice. These findings recommend limiting the intake of azo dyes and creating public awareness regarding their teratogenicity.
